# Brief intervention to prevent HIV, STI and unintended pregnancies: preliminary results of a feasibility study from the perspective of healthcare providers in Peru

**DOI:** 10.1186/s12913-021-07229-y

**Published:** 2021-11-12

**Authors:** Jean Pierre Jiron, Clara Sandoval, Juan Carlos Enciso, Ana Sofía De Vasconcelos, Karel Blondeel, Nataliia Bakunina, Galina Lesco, Igor Toskin, Rob Stephenson, Carlos F. Caceres

**Affiliations:** 1 Center for Interdisciplinary Studies in Sexuality, AIDS and Society, Universidad Peruana Cayetano Heredia, Lima, Peru; 2 Department of Sexual and Reproductive Health and Research, UNDP-UNFPA-UNICEF-WHO-World Bank Special Programme of Research, Development and Research Training in Human Reproduction (HRP), World Health Organization, Geneva, Switzerland; 3 Faculty of Medicine and Health Sciences, Ghent University, Ghent, Belgium; 4 Institute for Leadership and Health Management, I.M, Sechenov First Moscow State Medical University (Sechenov University), Moscow, Russian Federation; 5 National Resource Centre in Youth Friendly Health Services NEOVITA, Chisinau, Republic of Moldova; 6 Department of Systems, Population and Leadership, School of Nursing, University of Michigan, Ann Arbor, USA; 7 Center for Sexuality and Health Disparities, University of Michigan, Ann Arbor, USA

**Keywords:** Healthcare providers, Brief sexuality-related communication, Sexual health, Behavior change techniques

## Abstract

**Background:**

Brief interventions have proven to be valuable instruments for the treatment and care of clients with diverse health needs, due to their potential to impact both the individual and the population. In this regard, the Brief Sexuality-Related Communication (BSC) is presented as a viable and effective alternative for addressing sexual and reproductive health problems, assessing risk behaviors and motivating clients to generate behavioral change. Since health providers are key actors in treatment and prevention, it is essential to know their perceptions about the BSC intervention, as well as its acceptability in different contexts, with diverse client populations. Thus, the following paper reflects the findings of the perceptions and experiences of health providers in Peru from the first phase of the Feasibility study of a BSC intervention to prevent STIs and unintended pregnancies.

**Methods:**

This is the first phase of a multisite and multiphase study of the feasibility of a BSC intervention. We conducted twenty in-depth interviews (IDI) with health care providers (physicians, obstetricians, psychologists, nurses and peer counselors) recruited from three health care institutions in Peru: The Tahuantinsuyo Bajo Maternal and Child Center (CMI) and the San José Maternal and Child Center, both located in the capital city, Lima; and La Caleta Hospital located in Chimbote, northern coast of Peru. Participating health providers included those working at the HIV/STI Reference service and the family planning/reproductive health service. The IDI addressed three domains: 1) Acceptability of the BSC intervention; 2) Perceived willingness to implement the BSC intervention; and 3) Considerations for the Implementation of the BSC intervention.

**Results:**

Health providers expressed high acceptance of the BSC intervention, considering it as a useful and effective instrument to address sexual and reproductive health problems with all clients; however, some providers had some concerns about the real impact of the intervention to achieve significant behavior change. On the other hand, health providers showed high willingness to learn and implement the BSC intervention, affirming their commitment to learn new techniques and strategies that could allow them to improve their knowledge and the quality of their care. Health care providers consider it necessary to take into account the barriers that arise in the implementation of the BSC intervention, such as the structural limitations to access, the providers’ abilities to deliver the intervention effectively, and the participants’ reception of the intervention. Finally, providers consider it essential to establish the BSC intervention in a normative framework that allows it to receive the support of the health departments and eventually enforces implementation.

**Conclusions:**

Health providers consider the BSC intervention as an interesting and exciting behavioral intervention to deal with the sexual and reproductive health issues existing in different populations, and seemed highly willing to adapt and implement it, hoping that it become beneficial to all client populations to prevent HIV/STIs and unintended pregnancies.

## Introduction

It is essential for all people to achieve a state of comprehensive sexual health and well-being [1–3], and whether people can achieve this will largely depend on their access to comprehensive information about sexuality, their vulnerability to the adverse consequences of their sexual behavior, and access to high-quality sexual health care, as well as an environment that helps them affirm and promote their sexual health [4, 5].

In this regard, brief interventions are considered as informal counseling and information on certain types of harms and risks associated with risky behaviors, and aim to engage certain populations who are not yet ready for change. These brief interventions have become increasingly valuable for the treatment and care of clients, because of their low-cost benefits and proven effectiveness to handle a great diversity of public health issues [6–8]. Due to their potential to impact both the individual and the population, helping individuals to understand risk behaviors and motivating them to generate significant changes in their health care, brief interventions have been well received by health care providers and policy makers who consider them effective tools for prevention of risk behaviors in different public health arenas [9]. While brief interventions are based on a large scope of theoretical streams, the use of Motivational Interviewing principles is being more and more widely adopted to deliver brief interventions addressing health issues and achieving behavior change [10]. These brief interventions share general principles like: “*Feedback on existing risk situations and potential/real damages; the responsibility for the change rests with the person/patient; advice on practical strategies to reduce risky behaviors; a menu of options to help achieve behavior change; empathic delivery; and development of self-efficacy by healthcare providers”* [9].

The Brief Sexuality-related Communication (BSC) is defined in the WHO’s guidelines as an opportunistic, dynamic communication process between a patient and a trained health care provider that includes the addressing of diverse problems and difficulties related to sexuality and the promotion of sexual wellbeing, taking into account biological, psychological and social dimensions [11, 12]. The BSC aims to identify current and potential sexual issues and motivate those at risk to change their sexual behavior or maintain safe sexual behavior [6, 12, 13]. In addition, the BSC helps clients to re-evaluate their emotions and behavior for a better understanding of their sexual health, promoting critical reflection and setting clear goals, developing their capacity for self-regulation, and improving their autonomy and satisfaction, within a short 25-min session in primary care services [11, 12] The BSC is based on the theoretical dimensions of the “information, motivation and behavior” model [14, 15], and uses Motivational Interviewing (MI) techniques with a “client-centred” approach [6, 11], which respects clients’ ideas, feelings, expectations and values [13].

Healthcare providers are in a unique position to identify and intervene clients in risky situations and offer them a variety of options to address their sexual health issues [8]. Furthermore, clients often have confidence on healthcare providers’ expertise to address their concerns, and the information provided by healthcare providers plays a crucial role not only in the treatment, but also in the prevention of sexual health issues. In this way, health providers are the main actors in the delivery of health services, and it is very important that they are able to build a bond of trust and respect with clients that facilitates health care [16, 17]. At the same time, it is necessary for health providers to stay informed and trained in order to deal with the various issues that arise in sexual health [18–20].

When these conditions are not met, health providers can feel that their abilities are not adequate to screen and counsel clients with sexual health issues [8, 11]. That may lead to discomfort and insecurity when talking about sexual practices, particularly with populations with special sexual health needs [17, 19].

Although the effectiveness of brief behavioral interventions for the prevention of STIs/HIV has been shown, through different situations, for example, promoting positive attitudes and increasing condom use, reinforcing behavioral skills and establishing a non-threatening context, discussing concerns about sexuality, and offering empathetic understanding and unconditional support regarding clients’ behavior change [21], it is relevant to assess the feasibility of the BSC intervention in public facilities in lower-middle income countries, particularly when working with clients belonging to diverse vulnerable and stigmatized populations in various sociocultural contexts [4, 11, 12]. In this way, the BSC intervention generates new opportunities for health care providers to help their clients change behaviors that are relevant to their sexual health care [13, 22].

This report comes from the first phase of a feasibility study of the brief sexuality-related communication (BSC) intervention in Peru, as part of a WHO multiphase and multisite study [22], in which qualitative information was evaluated on the perceptions and attitudes of healthcare providers about the BSC intervention, and whether it would work in their daily practice offering sexual health services.

## Methods

### Study setting

Peru has an HIV epidemic concentrated on men who have sex with men (MSM), and transgender women (TW) [23, 24]. The estimated number of people over 15 years of age living with HIV in 2012 was 72,000 [25]. It has been estimated that 84% of new cases and 55% of prevalent cases occur in key populations [26]. HIV prevalence among MSM varies between 10 and 20% [24, 27]; and among TW it can reach 30% [28], with the very high risks for acquiring HIV in that group linked to high rates of sex work and drug use, themselves linked to extreme marginalization, social exclusion and violence. In Peru, homophobia and transphobia are still prevalent, and situations of discrimination and exclusion experienced by the lesbian, gay, bisexual, transgender and intersex (LGBTI) community are common in everyday life.

Likewise, in Peru the adolescent birth rate is estimated at 53 births per 1000 adolescents between the ages of 15 and 19 (2017–2018), and 12.6% of adolescents were mothers or became pregnant for the first time while in that age group [29]. Among pregnant adolescents, 64.2% did not seek or want their pregnancy at that time during 2019 [29]. Hence, the adolescent pregnancy rate has hardly changed in more than 20 years and, in relation to the national goal of reducing adolescent pregnancy by 20% in 2021, no progress has been made [30]. Adolescent motherhood continues to be the harshest expression of social injustice, poverty and vulnerability of women who come from rural areas, who are indigenous or come from the Amazon, and who struggle to have access to education and health, while live in a situation of poverty and exclusion [30, 31].

In response to these sexual and reproductive issues, the Peruvian state has been implementing the Technical Health Standard for Comprehensive Adolescent healthcare [32], which includes universal access to adolescent health and unrestricted medical care, covering a specialized package that includes sexual and reproductive health, physical, nutritional and mental health services. And specifically speaking about the prevention of child/adolescent pregnancy, the Technical Standard for Family Planning allows adolescents to access Sexual and Reproductive Health (SRH) services and contraceptive methods autonomously, without parental authorization [33]. However, some of the health providers still limit adolescents’ access to contraceptive methods [30]. Well-trained and sensitized healthcare providers with a human rights approach are needed to address adolescents’ diverse sexual and reproductive health needs.

### Procedures and participants

We chose Interpretative Phenomenological Analysis (IPA) to explore in detail the perceptions,attitudes and experiences of healthcare providers towards the BSC intervention in the SRH services (HIV, sexually transmitted infections [STI], pre-natal care, family planning, etc.), that work with MSM, TW, female sex workers (FSW), people living with HIV, adult women and adolescent females [34].

Twenty IDI were conducted between September 2018 and March 2019, with healthcare providers [physicians (2), obstetricians (12), psychologists (4) and peer counselors (2)] recruited from three health care facilities. The age range of healthcare providers was between 28 and 50 years and 18 of them were women, while only 2 were men (A physician and a peer counselor). Two of those facilities are maternal and child centers (CMI) in Lima with a status of primary care facility: the CMI Tahuantinsuyo Bajo, located north of Lima, and the CMI San José, located south of Lima. The third one is the La Caleta Hospital in the Ancash Region, located in the northern coast of Peru. All these facilities have HIV/STI referral centers (CERITS), where all clients are seen for the prevention and treatment of HIV & STIs, and also offer reproductive health services to adolescent and adult women (i.e. contraceptive methods, prenatal care and other). These facilities were chosen to carry out the study because they are part of the public health services and all have specific services where key populations receive care and this makes them ideal for finding suitable conditions and participants that can reflect the reality of the country health system.

Before initiating the IDI, the BSC intervention was described to healthcare providers and given brief details about the theoretical framework of the BSC intervention, the scope, its effectiveness, and the steps to implement it. For almost all healthcare providers, this was the first time they heard about BSC. The IDI addressed health care providers’ perceptions about the BSC intervention and explored providers’ knowledge to address clients’ specific needs in sexual health; sensitive issues to address with client populations; comfort working with key populations and discussing sexual health issues; strategies for addressing risk and prevention issues; acceptability of the intervention; barriers to delivering the intervention; and concerns about the intervention. These aspects were structured on three domains: 1) *Acceptability of the BSC intervention*, which seeks to explore the attitudes that health providers have towards the intervention, its perceived utility, and concerns and doubts about its impact; 2) *Perceived willingness to implement the BSC intervention*, which explores their willingness to participate in the training to learn the intervention, what aspects need to be reinforced during the training and their willingness to deliver the intervention; and 3) Considerations for the Implementation of the BSC intervention, addressing intervention feasibility, necessary conditions for its sustainability, barriers and difficulties for its implementation, and cultural adaptation of the intervention into the already-existing client-provider appointment were explored in interviews.

### Data collection and analysis

Every IDI lasted approximately 45–60 min, was audio-recorded and subsequently transcribed for analysis. Each transcript was reviewed for accuracy and any identifying information was removed. The IDIs were conducted by members of the research team in Peru. A code book was developed that helped structure the information recollected. During the coding process, several new codes and relational themes were identified, that allowed the creation of categories that were discussed and restructured by the research team members. We used Dedoose 8.3.35 software for the coding and analysis of interview transcripts, according to relational, familiarity and saturation criteria, applying an analytical framework to structure and guide the understanding of the outcomes and to identify the main categories of our study. Several meetings were necessary to discuss and restructure the analysis based on the findings and interpretations that later became key study findings.

## Results

### Acceptability of the BSC intervention

#### Provider attitudes towards the BSC intervention

Healthcare providers considered the BSC intervention to be a potentially effective tool that can contribute to the orientation and counseling of clients, especially with adolescents in search of a space where they can get answers about their sexuality and SRH without fear of being judged. Additionally, healthcare providers perceived it would be a very useful tool to reinforce the crucial role that they’re playing in the promotion and prevention of SRH issues.

*“It would be excellent because it is always important to handle this situation with young people who are quite disoriented; apart from the fact that they lack privacy, they have nobody to approach for help; they are afraid to ask parents, who also feel insecure to talk to them about these issues. So, who better than health professionals to offer them the confidence they need and help them with these issues? And to prevent pregnancy among adolescents, (a problem) that never ceases to grow”. (Interview 6, Obstetrician, La Caleta Hospital).*

In a manner of self-criticism, some providers manifested that despite having previously worked with different person-centered behavioral change techniques such as the 4C Counselling (Counselling, Compliance with treatment, treatment of Contacts and provision of Condoms) to control STIs and promotion of condom use, and the 5 Steps Model, for family planning butthey mentioned thathave not seen significant changes in the incidence of HIV and STI cases. Therefore, healthcare providers felt the urgent need to work with new strategies and techniques aimed at behavior change that are effective in the prevention of HIV and STIs.

*“Here we usually work with two types of counselling: The first is the best known, which is the 4C, you know, compliance, contact and condom counseling … mmm and the other is the 5 steps model, which are also on our normative guidelines that we have to take care of patients.” (Interview 2, Obstetrician, La Caleta Hospital).*

*“Of course, because by focusing on the person we can change behaviors; because so many strategies have been implemented, so many activities to prevent STIs and HIV … and the statistics continue to show new cases, then something happens and we can’t change anything fundamental, because the most difficult thing in someone is to achieve behavioral change”. (Interview 5, Obstetrician, La Caleta Hospital).*

#### Perceived utility of the BSC intervention

Healthcare providers considered that it would be very useful to address directly with clients the possibility of improving their sexual and reproductive health care by using innovative strategies. Providers also noted that clients in general would greatly benefit from this intervention because they would perceive the empathy and interest from providers to help them solve their sexual health issues and improve their wellbeing.

*“Yes, I think so, because they are going to feel that they care about you, and that you want to do something for him or her and that you really listen to them. People come here because they want to be heard”. (Interview 18, Peer counselor, CMI San José).*

*“Of course, because when you open it up, the client talks to you about other topics that are also suddenly generating anxiety at that moment and they are a little more liberated from suddenly believing that what they were thinking was something strange and not. Talking about sexuality is part of ourselves and we are also a consequence of our sexuality”. (Interview 3, Psychologist, La Caleta Hospital).*

In this way, health care providers manifested that the BSC intervention will be of great help to deal more fully with risky situations. Furthermore, they stated that the intervention addresses comprehensive care, including other personal and emotional aspects. This would reinforce prevention work at a deeper and more complex level.

“*… It will help us greatly (to promote) changes in our patients’ sexual behaviors, which is what we also seek with our counseling, to change or help prevent risk behaviors more than anything, offering them the knowledge and helping them avoid risky behaviors”. (Interview 13, Obstetrician, CMI Tahuantinsuyo Bajo).*

*“It would be like doing prevention work in many aspects, because one thing they come here for is to rule out if they have any STIs. So, it would be to prevent with comprehensive information, because not only are we going to give them information about STIs, but we are also going to talk about the personal part, about controlling your drive, your emotions, and about your self-esteem, so that then they use protection and can take care. So that would be preventive, it seems very good to me”. (Interview 20, Psychologist, CMI San José).*

#### Concerns and doubts about BSC Intervention’s impact

However, healthcare providers stated that the intervention’s impact would likely be dependent on who delivers it and how it is delivered, so that it can have a positive impact on the clients, helping them to recognize strengths and weaknesses and to generate concrete behavioral changes (to improve) their sexual health.

*“I think it would be very useful, because depending on the counseling, they will apply it in their lives, and that will help them to know what strengths they have that they did not even know about, or that they had not even perceived they had. And weaknesses that they were not conscious about either”. (Interview 18, Peer counselor, CMI San José).*

On the other hand, health providers expressed various doubts and concerns about the real impact the intervention can make to tackle sexual health issues. They comment on clients who re-engage in the same risk behaviors, stating that a single session of the intervention can’t be sufficient to generate significant behavioral change, because it is a long and constant process in which the health provider accompanies the client. In addition, providers manifested that clients’ final decisions regarding their sexual health issues are also influenced by their environment, which is not under the providers’ control.

*“Well, it is assumed that, if we are going to take some time to work on a single topic, it is because we have to do it, and (that decision) will be effective to (ensure we) achieve something little by little, to make a change, because changes among people do not happen overnight, and because we can say one thing, but when we go home, father, mother and the whole environment may influence our decisions (to do otherwise)”. (Interview 14, Obstetrician, CMI Tahuantinsuyo Bajo).*

*“In the case of patients who have an STI, we also try to follow the same procedure a bit, but we must insist on why they had an STI, and what can happen if they continue with the same behaviors. Obviously what counseling is looking for is a change in behavior, but we know that it will not be achieved with counseling only or in a few months. Rather it is a whole process that takes some long time”. (Interview 15, Psychologist, CMI Tahuantinsuyo Bajo).*

### Perceived willingness to implement the BSC intervention

#### Willingness to perform the BSC intervention

Health care providers expressed their willingness to implement the BSC intervention by saying that it can benefit both clients and themselves as long as the methodology to be implemented meets the criteria of confidentiality, privacy and respect while being focused on the welfare of the client.

*“… If the intervention continues to meet the criteria of confidentiality, privacy, then, (if) used by health staffing search of the same results, it will be beneficial for the health provider and the patient”. (Interview 15, Psychologist, CMI Tahuantinsuyo Bajo).*

Similarly, healthcare providers remained open to learn new methods and techniques that can help them address sexual health issues with their clients, and they are happy to receive this support from various health institutions.

*“If there are changes for the good of the population, welcome. We are happy to support them, because now they are with the WHO, but the idea would be for the Ministry of Health to take it, and if they are also going to support us with some logistics, in good time. We should not say no to the changes that are coming”. (Interview 14, Obstetrician, CMI Tahuantinsuyo Bajo).*

#### Willingness to participate in the training for the BSC intervention

Likewise, healthcare providers considered necessary and essential that they are able to carefully review the BSC intervention in depth. Therefore, providers stated that training is crucial for the team to learn, evaluate and adapt the intervention. In addition, healthcare providers reported that it would be very useful for them to learn, as well as to reinforce the topic of social skills.

*“Well, any project or program that you want to logically implement, you have to first receive the training to implement it. (Then you) present it as a group in a technical meeting, with the whole team, and I think the whole team would also agree. With anything to improve the consultation and patient care I think there would be no problem”. (Interview 13, Obstetrician, CMI Tahuantinsuyo Bajo).*

*“First is the issue of what the BSC intervention is (or consists of). Everything that corresponds to the technical part, strengthening the skills issue as well, in the search for information about the different populations that we deal with, it seems important to me, how to reach that client who is different”. (Interview 5, Obstetrician, La Caleta Hospital).*

#### Necessary aspects to reinforce in the training of the BSC intervention

For the training sessions, health providers suggested that concepts related to gender, gender identity, and other issues that allow them to address the specific needs of each of the client populations should be reinforced.

*“There should be much more in itself, addressing gender issues, gender identity, so that we have much more to talk about when intervening the population, we manage”. (Interview 10, Obstetrician, CMI Tahuantinsuyo Bajo).*

Similarly, healthcare providers considered vital to reinforce the strategies for approaching issues of sexual rights, the use of contraceptive methods, the resolution of partner conflicts and especially the issue of violence, since these are the cases they frequently deal with.

*“Sexual rights, especially the issue of violence, the issue of sexual rights, the issue of the couple relationship, the issue of contraceptive methods”. (Interview 8, Obstetrician, La Caleta Hospital).*

### Considerations to implement the BSC intervention

#### Feasibility of the BSC intervention

Healthcare providers stated that human and material resources as well as training of the team of health professionals is vital for intervention implementation. Taken together it will play a crucial role in the feasibility of the BSC intervention.

*“Well, I believe that as long as there are human resources and the necessary materials to implement it and maintain it, then we will all be able to do it”. (Interview 13, Obstetrician, CMI Tahuantinsuyo Bajo).*

In order to perform the intervention in the best possible way, providers mentioned that the interactions between healthcare providers and clients must met certain conditions by which the clients feel comfortable, safe and confident enough to address their sexual health issues. That is why healthcare providers pointed out that will depend on their skills to apply the principles of the intervention and their capacity to reach clients with empathy, compassion and respect.

*“It all depends on who speaks to them... rather, the people who are going to give this type of intervention must be suitable people, who have that freedom, who know how to gain empathy, who know how to reach the patient, who have those qualities”. (Interview 4, Obstetrician, La Caleta Hospital).*

*“Many times they shy away from telling you many things, or you do not give them confidence because of the tone of voice, or sometimes how you ask, because they may think that you are judging them”. (Interview 7, Obstetrician, La Caleta Hospital).*

#### Conditions for the sustainability of the BSC intervention

Providers stated that for the intervention to be sustainable, it will be necessary to build communication networks between health services and those responsible for making decisions in healthcare facilities. It is vital that a commitment is maintained by decision makers to guarantee the supply of necessary material conditions, as well as the availability of staff to carry out the intervention in health services.

*“(We) try to get others involved … one of the things we do in this HIV and STI REFERENCE CENTER (CERITS), despite the fact that we do not have an assigned population, and that we are not a health network, is to share the information we have, all the time. We are personally committed to transfer to other health networks any training we receive”. (Interview 5, Obstetrician, La Caleta Hospital).*

Most healthcare providers manifested that, although they may positively accept the intervention, its implementation will depend on the decisions made by the heads and leaders of the health services and, at times, this can represent a barrier.

*“That also depends on the head; it is a bit complicated … It depends on the Director, because sometimes a director comes up with a new idea, the other director says no, and many colleagues abide by that”. (Interview 9, Obstetrician, CMI Tahuantinsuyo Bajo).*

#### Barriers and difficulties to implement the BSC intervention

However, healthcare providers manifested that there are some structural barriers that represent difficulties in care, related to reduced spaces and inadequate infrastructure of the health facility, which hinder confidentiality; inflexible schedules, and staff shortages, which generate long waiting times for clients.

*“We don’t have the ideal facility; in other words, space is a limitation ... another is the time we have to offer counseling”. (Interview 7, Obstetrician, La Caleta Hospital).*

*“Barriers include time - because patients do not have much time; and also a waiting list; logically that will cause discomfort. Another potential barrier is the rejection of patients, who suddenly do not want to receive the necessary guidance or who are in a hurry, basically that”. (Interview 13, Obstetrician, CMI Tahuantinsuyo Bajo).*

#### Providers’ perceived self-efficacy to implement the BSC intervention

Healthcare providers felt their skills to adapt the BSC intervention to their daily activities could be tested. In some cases, they perceived that not having had knowledge before about the BSC intervention could be a limitation to carry it out. However, they stated that they are willing to learn the intervention to benefit their clients and address their sexual health issues in the best possible way.

*“Maybe at the beginning yes, more than anything on the part of the health personnel, as in everything, it is a process, and for the health personnel also if it is a new methodology, a new strategy, it will take a little bit of time to adapt, but if it is so beneficial and effective, it is okay to implement it”. (Interview 15, Psychologist, CMI Tahuantinsuyo Bajo).*

Although the healthcare providers indicated that they maintain a positive disposition to carry out the BSC intervention, as it was a short session, they presented certain concerns regarding their abilities to be able to work on all the issues that clients need in that short session.. Meanwhile, there are clients for whom longer visits would not represent a problem, healthcare providers still recommended to keep visits as short as possible.

*“Sure, as long as it doesn’t last long. Because, when the visit takes much longer, some clients may feel uncomfortable…, but it is very diverse… on the one hand there are patients who want to listen to you and want to continue talking; on the other hand, there are those who come in a hurry and have to go to work immediately. That really depends on each patient”. (Interview 13, Obstetrician, CMI Tahuantinsuyo Bajo).*

## Discussion

Our findings about perceptions of the BSC intervention among health care providers in Peru have shown that the BSC intervention has been perceived as a potentially effective instrument for sexual and reproductive health care, aiming to assist the clients to reflect on the reasons for their risk behaviors, re-evaluate their emotions and understand their sexual health better, motivating them to change their behaviors [11]. In addition, health care providers highlight the potential benefit of improving their ability to establish an empathic interaction with clients, helping them set goals and develop their autonomy, while simultaneously the BSC intervention gives the client greater satisfaction and security to face their sexual health concerns [12]. Moreover, due to its innovative nature, the BSC intervention is seen by health providers as a groundbreaking opportunity to address various sexual and reproductive health problems in different populations, linked to the high rates of unintended pregnancies in recent years [29, 30], and the multi-layered vulnerability faced by key populations regarding HIV and STIs [24, 28, 31]. It is important to clarify, however, that our findings here represent providers’ perceptions about BSC after getting explanations of the principles of BSC, but before being trained on and directly exposed to this intervention.

From an instrumental viewpoint, it is considered essential for health care providers to remain up-to-date with new interventions and techniques aimed at changing behaviors for HIV/STI prevention [18–20]. In fact, health care providers consider that being trained to implement the BSC intervention will allow them to address different needs in sexual and reproductive health, in line with findings in other studies [6, 11].

Although the BSC intervention concept is naturally appealing to health care providers due to the flexibility it implies to approach sexual health issues, providers also express some doubts about the real impact it may have on clients’ behaviors. Some providers expressed that addressing sexual health issues is part of a much larger and complex process that can hardly be addressed in a single session; or added that the various situations of inequality and vulnerability in which many clients find themselves must be considered. While the BSC intervention has been designed to not only help patients make and enact behavioral change decisions, but also to make them feel more in control of their health through positive change [11–13], it is true that external factors play a key role, and they should be addressed during the training.

Regarding their willingness to learn and implement the intervention, health care providers expressed their commitment to learn in detail the guidelines of the BSC intervention and adapt it to their medical consultations, since they consider that strengthening their skills, would be very useful for them at a professional level, as well as for clients, offering them much more effective alternatives to face their sexual health issues. While we feel this enthusiasm is positive, we also realize that providers normally welcome any training that offers to help them innovate and improve their practice, and have to observe how this translates into actual commitment to change in real life [16, 18, 20, 21].

Health care providers also state that it is necessary to address strategies to approach vulnerable populations in the BSC intervention trainings, and that they would like to learn much more about aspects such as gender identity, human rights, intimate partner violence, sexual abuse, abortion, among others, since those are some of the sexual and reproductive health problems most frequently encountered when seeing clients. This reflects a perspective expressed by providers across several studies in Peru [28, 30, 31].

Regarding the necessary considerations to be able to effectively implement the BSC intervention, health care providers stated that it is essential to have the logistical resources that allow them to deliver the intervention, including appropriate training. Moreover, providers emphasize that, in order to ensure the sustainability of this intervention, it must be incorporated into a national regulation that provides legitimacy and turns it into a regular tool to be used in sexual and reproductive health services [32, 33].

Clearly, there are various structural barriers within health facilities that represent a challenge for the implementation of this intervention, including the health facility infrastructure, inflexible schedules, lack of health personnel and limited times to offer the intervention. Some providers indicate that their capacity to adapt and execute the intervention in their regular work with clients will be tested [19, 20]. While most of them have expressed a high desire to implement the intervention, they acknowledge that it does not ensure implementation with a high degree of fidelity, which can affect effectiveness. This emphasizes the need for a specific policy that is enforced in order to make the implementation of the BSC intervention possible.

Finally, it is crucial for health providers to consider the variability of clients’ reactions to the offer of an in-depth, more interactive intervention. While some might welcome it, others might be bothered by the extra time it demands, or find it intrusive and unwarranted [17, 19]. Such feedback is important for any effort to adapt the BSC intervention to the specific needs of the populations served by public HIV/STI and SRH services in Peru, and possibly in many parts of Latin America.

## Conclusions

Healthcare providers expressed high acceptance of the BSC intervention as a potentially effective instrument to address sexual and reproductive issues with diverse client populations. These perceptions about the BSC intervention were expressed after it was described to them, but before they had been trained and exposed to it. Many health providers indicated they were willing to implement the BSC intervention, and felt very motivated to receive the intervention training, considering that it could become very helpful to improve their skills and achieve better results when addressing their clients’ sexual health concerns. However, some expressed skepticism about its capacity to elicit significant behavioral changes and lead to improved clients’ sexual health given its brevity. It is also necessary to consider the various aspects that can hinder the implementation process of the BSC intervention, including the structural conditions of health facilities, the ability of health providers to deliver the intervention with fidelity, and some clients’ reaction to the offer of a more in-depth, more comprehensive interaction that they find too lengthy or simply unwarranted. In conclusion, these findings portray a generally positive disposition among providers, in spite of some challenges, and will contribute in the design and implementation of the subsequent phases of this study.

## Data Availability

The data sets generated and analyzed during the current study are not publicly available because due to their qualitative nature, they were processed to draw conclusions from unstructured and heterogeneous data that are not expressed in a numerical or quantifiable way; but are available from the corresponding author on reasonable request.
