# Correction to: Brief intervention to prevent HIV, STI and unintended pregnancies: preliminary results of a feasibility study from the perspective of healthcare providers in Peru

**DOI:** 10.1186/s12913-021-07417-w

**Published:** 2021-12-28

**Authors:** Jean Pierre Jiron, Clara Sandoval, Juan Carlos Enciso, Ana Sofía De Vasconcelos, Karel Blondeel, Nataliia Bakunina, Galina Lesco, Igor Toskin, Rob Stephenson, Carlos F. Caceres

**Affiliations:** 1 Center for Interdisciplinary Studies in Sexuality, AIDS and Society, Universidad Peruana Cayetano Heredia, Lima, Peru; 2 Department of Sexual and Reproductive Health and Research, UNDP-UNFPA-UNICEF-WHO-World Bank Special Programme of Research, Development and Research Training in Human Reproduction (HRP), World Health Organization, Geneva, Switzerland; 3 Faculty of Medicine and Health Sciences, Ghent University, Ghent, Belgium; 4 Institute for Leadership and Health Management, I.M, Sechenov First Moscow State Medical University (Sechenov University), Moscow, Russian Federation; 5 National Resource Centre in Youth Friendly Health Services NEOVITA, Chisinau, Republic of Moldova; 6 Department of Systems, Population and Leadership, School of Nursing, University of Michigan, Ann Arbor, Michigan USA; 7 Center for Sexuality and Health Disparities, University of Michigan, Ann Arbor, Michigan USA

**Correction to: BMC Health Serv Res 21, 1225 (2021)**

**https://doi.org/10.1186/s12913-021-07229-y**

Following the publication of the original article [1], some information is missing in the Funding section and the author needs to add a Disclaimer section.

The updated Funding and Disclaimer sections are given below.

**Disclaimer**

Some of the authors are present staff members of the World Health Organization. The authors alone are responsible for the views expressed in this article and they do not necessarily represent the views, decisions or policies of the institutions with which they are affiliated.

**Funding**

This work was funded by the UNDP-UNFPA-UNICEF-WHO-World Bank Special Programme of Research, Development and Research Training in Human Reproduction (HRP), a co-sponsored programme executed by the World Health Organization (WHO).

The original article [1] has been corrected.
